# Chimeric epitope vaccine against *Leptospira interrogans* infection and induced specific immunity in guinea pigs

**DOI:** 10.1186/s12866-016-0852-y

**Published:** 2016-10-14

**Authors:** Xu’ai Lin, Guohui Xiao, Dongjiao Luo, Liangliang Kong, Xu Chen, Dexter Sun, Jie Yan

**Affiliations:** 1 Department of Medical Microbiology and Parasitology, School of Medicine, Zhejiang University, 866 Yuhangtang Road, Hangzhou, 310058 People’s Republic of China; 2 Basic Medical Microbiology Division, State Key Laboratory for Diagnosis and Treatment of Infectious Diseases, School of Medicine, Zhejiang University, Hangzhou, 310058 People’s Republic of China; 3 Department of Neurology and Neuroscience, New York Presbyterian Hospital and Hospital for Special Surgery, Cornell University Weill Medical College, New York, NY 10021 USA

**Keywords:** Leptospira, Outer membrane protein, Multi-epitope, Vaccine, Cross protection

## Abstract

**Background:**

Leptospirosis is an important reemerging zoonosis, with more than half a million cases reported annually, and is caused by pathogenic *Leptospira* species. Development of a universal vaccine is one of the major strategic goals to overcome the disease burden of leptospirosis. In this study, a chimeric multi-epitope protein-based vaccine was designed and tested for its potency to induce a specific immune response and provide protection against *L. interrogans* infection.

**Results:**

The protein, containing four repeats of six T- and B-cell combined epitopes from the leptospiral outer membrane proteins, OmpL1, LipL32 and LipL21, was expressed and purified. Western blot analysis showed that the recombinant protein (named r4R) mainly expressed in a soluble pattern, and reacted with antibodies raised in rabbit against heat-killed *Leptospira* and in guinea pigs against the r4R vaccine. Microscopic agglutination tests showed that r4R antisera was immunological cross-reactive with a range of Chinese standard reference strains of *Leptospira* belonging to different serogroups. In guinea pigs, the r4R vaccine induced a Th1-biased immune response, as reflected by the IgG2a/IgG1 ratio and cytokine production of stimulated splenocytes derived from immunized animals. Finally, r4R-immunized guinea pigs showed increased survival of lethal *Leptospira* challenges compared with PBS-immunized animals and tissue damage and leptospiral colonization of the kidney were reduced.

**Conclusions:**

The multi-epitope chimeric r4R protein is a promising antigen for the development of a universal cross-reactive vaccine against leptospirosis.

**Electronic supplementary material:**

The online version of this article (doi:10.1186/s12866-016-0852-y) contains supplementary material, which is available to authorized users.

## Background

*Leptospira interrogans* is the causative agent of zoonotic leptospirosis, which affects humans in both developing and developed countries. It is a major public health problem in many areas, particularly after floods and heavy monsoons. Globally, it has is estimated that 0.1 to 1 per 100,000 people living in temperate climates are affected each year, with the numbers increasing to ten or more per 100,000 people living in tropical climates (according to World Health Organization, The Global Burden of Leptospirosis). In China, between 2002 and 2007 approximately 1500 people were infected with *L. interrogans* of which 50 died [1]. People are generally infected by contaminated water or soil sources through exposure of wounds in the skin or through exposure of the mucosal layers [2]. The symptoms following infection can vary from a mild febrile illness to more severe icteric disease, which is characterized by potentially fetal renal and liver failure [3].

Despite the constant progress of antimicrobial therapeutics, vaccination is still expected to be the most effective method to protect people that come in close contact with infected animals or environments. Both inactivated and attenuated vaccines have been used in animals or even in humans. However, these vaccines are associated with high rates of side-effects, such as aches and anaphylaxis, and they confer only short-term serovar-specific immunity [4, 5]. Currently, studies on developing leptospirosis subunit vaccines are particularly focused on bacterial motility, lipopolysaccharides (LPSs), lipoproteins, outer-membrane proteins (OMPs) and potential virulence factors. However, the protective efficacy of these candidates in experimental animals is low (40–75 %) [6]. Thus far, the most promising subunit vaccine candidates are the Lig proteins, which have been shown to confer high-level protection, approaching 100 % in mice and hamsters [7–10]. However, whether the Lig proteins are able to elicit cross-protective immunity to a range of serovar remains to be determined, since the similarity of the amino acid sequence of this protein between different *Leptospira* spp. is 70–100 % [6]. Since the currently available vaccines are characterized by a short duration of immunity, side-effects or serovar specificity, it is important to develop a universal leptospirosis vaccine with high efficiency and low toxicity.

For this purpose, we screened different *Leptospira* serotypes for conserved surface-exposed antigens. Bacterial outer membrane proteins (OMPs) are a major target of the immune system in a variety of infectious diseases and they have been suggested as candidates for diagnosis and immunization [4]. In *Leptospira*, three classes of these OMPs have been identified: 1) lipoproteins, which are the most abundant class and include proteins such as LipL32, LipL41 and LipL21 [11–13]; 2) trans-membrane proteins, which include OmpL1 [14]; and 3) peripheral membrane proteins, which include LipL45 [15]. In our previously studies we have identified that OmpL1, LipL32 and LipL21 are expressed in all the pathogenic strains of 15 serovar/serogroup standard reference strains of *Leptospira* in China [16–18]. Furthermore, production of these proteins was down-regulated (about 10–50 fold) in host-adapted *Leptospira* [19], suggesting that these proteins might not be involved in the pathogenesis of *Leptospira* after it gains entry into the mammalian host, which indicates that these OMPs are safe vaccine candidates.

*Leptospira* contains over 200 pathogenic serovars, which are divided over 25 serogroups. The diversity of *Leptospira* is also reflected in its OMPs, which are genetically diverse between different serogroups [16–18]. In addition, given the large size of OMPs, conserved epitopes might also be sterically blocked. For these reasons, an OMP vaccine might still provide only minimal protection against a heterologous *Leptospira* infection. Alternatively, conserved functional OMP epitopes could be used for the development of a peptide vaccine [20]. However, it is difficult to develop an epitope-based peptide vaccine for human clinical trials, because of the technical limitations or economic reasons in synthesizing large quantities of highly purified peptides. Our strategy to overcome these difficulties was to develop a multi-epitope chimeric protein that is composed of the immunodominant epitopes from the OMPs OmpL1, LipL32 and LipL21, which are separated by a tetraglycine linker. The selected epitopes consisted of both B-cell epitopes, which are essential for protective antibody response, and T-cell epitopes, which serve to induce a cellular immune response.

## Results

### Expression, purification and immunogenicity of the chimeric r4R protein

**Fig. 1:**
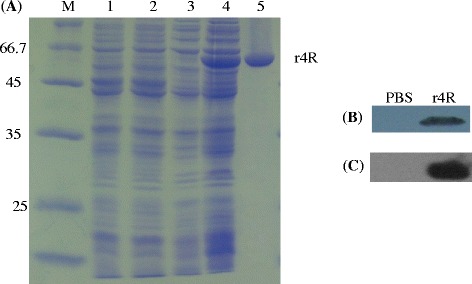
Characterization of the expressed and purified chimeric r4R protein. **a** SDS-PAGE analysis of the expression and purification steps of the r4R protein. Pellet (lane 1 and 3) and supernatant (lane 2 and 4) fractions of lysates from *E. coli* BL21 (DE3) cells containing the empty vector pET28a (lane 1 and 2) or pET28a-4R (lane 3 and 4) were electrophoresed in a 10 % SDS-PAGE gel. M indicates the protein ladder. Lane 5 contains the purified recombinant 4R protein. **b** Anti-*Leptospira* Western blot analysis. The purified chimeric r4R protein was run on a SDS-PAGE gel and transferred to a PVDF membrane. Serum from heat-killed *L. interrogans* strain Lai immunized rabbits was used as a primary antibody to detect r4R. Serum from PBS injected rabbits was used as control. **c** Anti-r4R Western analysis. The purified chimeric r4R protein was run on SDS-PAGE gel and transferred to a PVDF membrane. Serum from guinea pigsimmunized with PBS (negative control) or chimeric protein, were used as primary antibody

Based on the sequences of screened epitopes [21, 22], a gene fragment containing six selected epitope sequences was synthesized (Additional file 1) and cloned as four consecutive repeats in an expression vector, thereby forming a single chimeric multi-epitope encoding gene. The protein was successfully expressed in *Escherichia coli* BL21 (DE3) and purified. SDS-PAGE results showed that the full-length 64 kD protein was induced by IPTG and present in the soluble fraction of lysed *E. coli* BL21 (DE3) cells (Fig. 1a). Furthermore, SDS-PAGE analysis of the purified protein under native conditions showed a single band at 64 kD (Fig. 1a). The yield of the protein purification process was approximately 10.2 mg of protein from 1 l culture. The protein sample was passed through a Detoxi-Gel column, which contains a modified polymyxin prime B (PMB) that removes lipopolysaccharide (LPS) contaminations. No residual LPS was detected in the protein extract using a limulus test, which has a detection sensitivity of 10 pg (data not shown). The purified recombinant protein was named r4R.

To evaluate reactivity of r4R with antibodies raised in rabbits against heat-killed *L. interrogans* strain Lai, Western blot analysis was performed. Antibodies raised in rabbits against heat-killed *L. interrogans* were showing a strong interaction with r4R (Fig. 1b), indicating that the epitopes of r4R are present on the surface of *L. interrogans*. To evaluate the immunogenicity of r4R, guinea pigs were immunized with r4R and the collected serum analyzed. Western analysis showed that the chimeric multi-epitope r4R protein was able to induce an antigen-specific humoral immune response (Fig. 1c). The immunodiffusion titer of the antisera against the chimeric r4R protein was 1:8.

**Fig. 2:**
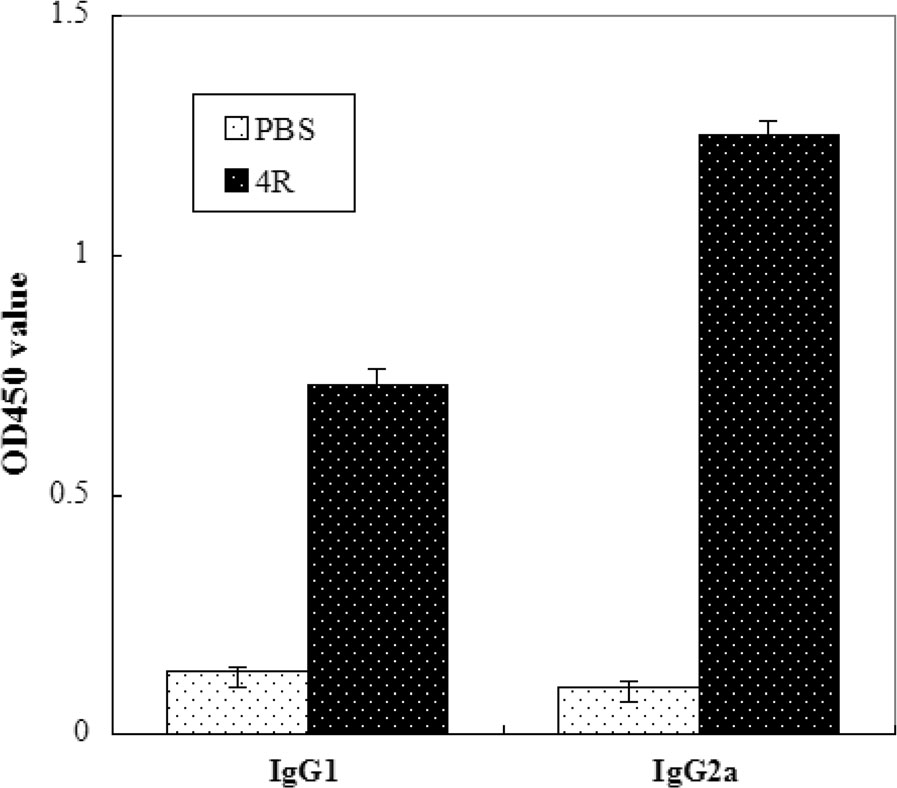
Response of IgG subclasses on the chimeric protein. IgG subclasses induced by the chimeric protein in guinea pigs were detected by ELISA

To evaluate the polarity of the immune response against r4R, antigen-specific IgG subtype (IgG1 and IgG2a) responses in the sera of vaccinated and control guinea pigs were measured by ELISA. The results showed that although the levels of both IgG1 and IgG2a were increased in the sera of animals immunized with the recombinant protein compared with the control sera from PBS treated animals, the level of IgG2a was higher than that of IgG1 (Fig. 2). The elevated IgG2a/IgG1 ratio points to a Th1 polarized immune response in the r4R-immunized guinea pigs. To determine the cross-reactivity of serum obtained from r4R-immunized guinea pigs, MAT analysis was performed using the most commonly encountered serogroups of *L. interrogans* in China, namely Icterohaemorrhagiae, Pomona, Canicola, Hebdomadis, Autumnalis, Grippotyphosa and Australis. Serum from the r4R-immunized guinea pigs was able to cross-agglutinate each of the *L. interrogans* serogroup-specific standard reference strains with titers ranging from 1:80 to 1:320 (Table 1). These results indicate that r4R is an immunological cross-reactive protein, which is important for the coverage of a leptospiral vaccine.

**Table 1:** MAT analysis of sera from r4R-immunized guinea pigs against various *L. interrogans* serogroups

Serogroup	Strain	MAT titer (1:N)	Normal
Icterohaemorrhagiae	56,601	320	-
Grippotyphosa	56,609	80	-
Autumnalis	56,606	160	-
Pomona	56,608	160	-
Australis	56,607	80	-
Hebdomadis	56,610	160	-
Canicola	56,603	80	-
Sejroe	56,635	80	-

### Lymphocyte proliferation and cytokine production in response to chimeric r4R protein

**Fig. 3:**
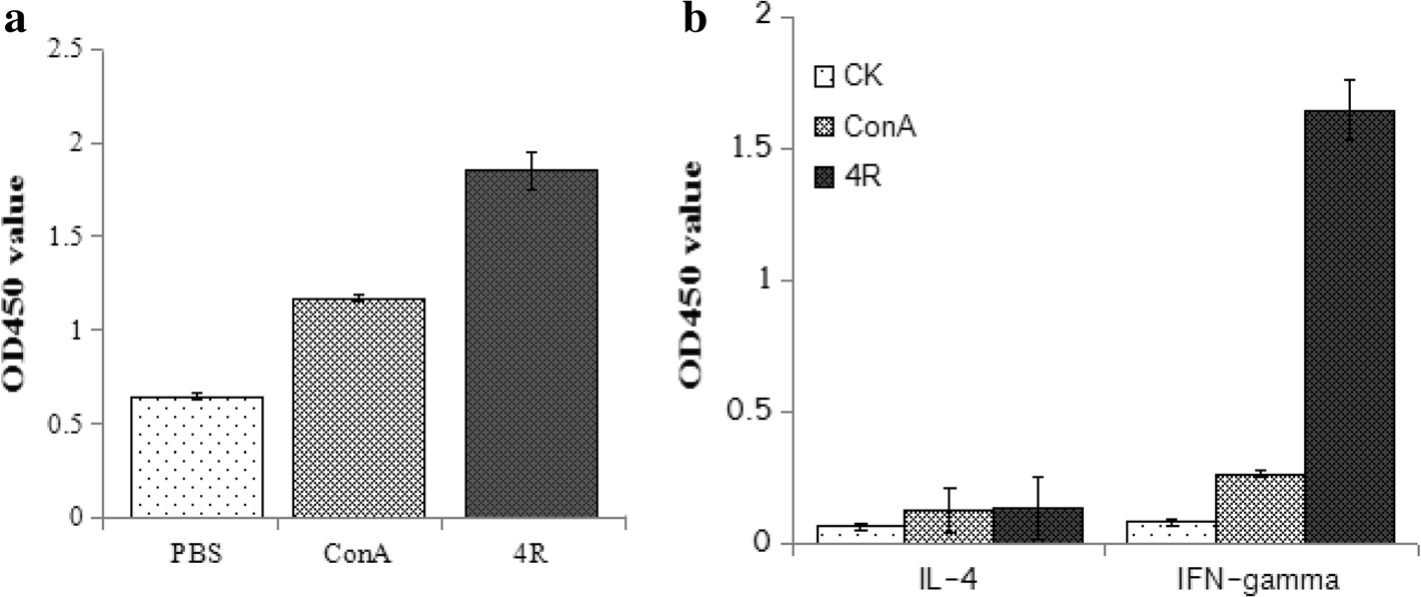
Proliferative and immune responses of splenocytes immunized with chimeric protein. Guinea pigs were immunized with the chimeric r4R protein. Ten days after the last immunization, the splenocytes were isolated, stimulated with the r4R protein and cultured for 72 h. **a** Splenocyte proliferation analysis of PBS, ConA and r4R stimulated splenocytes. **b** ELISA measurements of IL-4 and INF-γ secreted by pooled control, ConA and r4R stimulated splenocytes and lymphocytes in the culture supernatants

Lymophocytes from r4R-immunized guinea pigs were stimulated ex vivo with the r4R protein and analyzed for proliferation and cytokine production. Lymphocytes stimulated with r4R showed a significantly higher proliferation response compared with the controls (Fig. 3a). The supernatant of the r4R stimulated lymphocytes was analyzed for IFN-γ and IL-4 cytokine levels by ELISA. IFN-γ production was 20-fold higher in lymphocytes that were isolated from animals immunized with the chimeric r4R protein and subsequently stimulated with r4R compared with the controls (Fig. 3b). No significant differences in IL-4 cytokine production were observed (Fig. 3b), indicating again that r4R specifically stimulates a Th1-biased immune response.

### Protective efficacy of the chimeric r4R protein vaccine

**Fig. 4:**
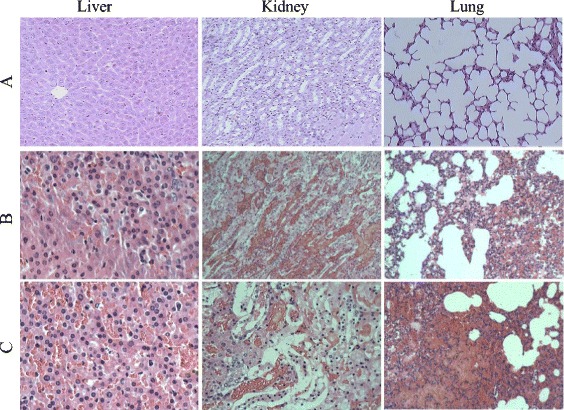
Histopathological effects of *Leptospira* challenges on immunized guinea pig tissues. Representative images of liver, kidney and lung tissues of healthy guinea pigs (**a**) or guinea pigs immunized with the chimeric r4R protein (**b**) or PBS (**c**) and challenged with *L. interrogans* strain Lai. Tissues were stained with hematoxylin and eosin

**Fig. 5:**
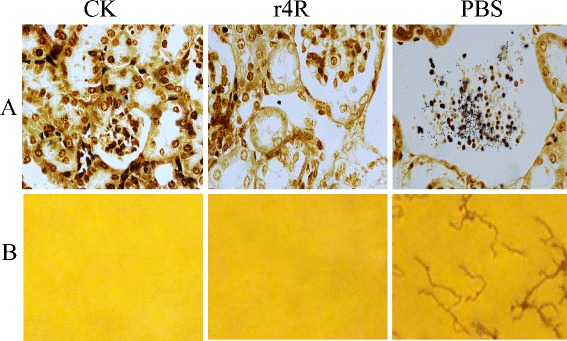
Colonization of *L. interrogans* in chimeric r4R immunized guinea pigs. Representative images of kidney lysates (**a**) or urine samples (**b**) that were collected from healthy control guinea pigs (CK) of guinea pigs immunized with r4R or PBS and subsequently challenged with *L. interrogans* strain Lai. All samples were strained with the Fontana silver staining method and observed using an optical microscope

To investigate whether the chimeric r4R protein is able to induce protective immunity against a lethal *L. interrogans* infection, guinea pigs were immunized and challenged with *L. interrogans* strain Lai, for which the LD_50_ was determined at 1 × 10^8^. PBS-immunized guinea pigs challenged with a 2 × LD_50_ died within 8–10 post-infection, while 80 % (4/5) of the r4R-immunized guinea pigs survived challenges with this dose. Liver, kidney and lung tissues of the challenged PBS-immunized guinea pigs showed severe leptospirosis symptoms, including necrosis (Fig. 4). In the kidney and liver, severe lesions were observed, including hemorrhages, while histopathological lesions in the kidney showed renal tubular necrosis with occasional polymorphonuclear cell infiltration and loss of normal liver architecture. In contrast, liver, kidney and lung tissues of r4R-immunized animals showed only mild edema symptoms (Fig. 4), and there was little or no evidence of lesion formation. Furthermore, in Fontana silver-stained kidney samples, no *Leptospira* were detected in the r4R-immunized animals, while many *Leptospira* were detected in the samples from PBS-immunized animals (Fig. 5a). In addition, after incubation of the lysed kidney samples in EMJH medium at 30 °C for 28 days, floccules were observed in the EMJH medium by tungsten lamp analysis of the PBS-immunized guinea pig samples, while none were detected in the samples from r4R-immunized animals (data not shown). Finally, no leptospires were detected in the urine samples of r4R-immunized guinea pigs, while some were encountered in the urine samples of PBS-immunized animals (Fig. 5b). Together, these results indicate that immunization of guinea pigs with the chimeric multi-antigen r4R protein provides protection of the animals against lethal challenges of *Leptospira* and prevents severe tissue damage.

## Discussion

*L. interrogans* is a bacterial pathogen that causes widespread disease in both animals and humans. Leptospirosis poses a significant public health problem since it is potentially fatal due to the damage it can cause to multiple organs, including the liver, lungs, kidney and brain [23]. Leptospirosis is prevalent throughout China, except for some of the Western provinces (Xinjiang, Xizang, Qinghai, Gansu and Ningxia). Particularly hard hit are the regions in the Yangtze River basin, such as the provinces Sichuan, Hubei and Hunan and the area south of the Five Ridges. The most commonly encountered *L. interrogans* serogroups in China are Icterohaemorrhagiae, Pomona, Canicola, Hebdomadis, Autumnalis, Grippotyphosa and Australis [5]. The currently available and widely used vaccine against leptospirosis is a multivalent inactivated whole-cell vaccine. However, this vaccine shows high side-effects and elicits little cross-protection against strains from different serovars. Therefore, a new effective and safe vaccine that is able to provide cross-protection of at risk humans or animals is urgently needed.

Currently, vaccine research against *Leptospira* is focused on the discovery of cross-reactive conserved antigens that are able to provide long-term protection against a broad range of *Leptospira* spp. [24]. It is well established that OMPs show a high degree of conservation in *Leptospira* spp.. Furthermore, they are immunodominant antigens in both humans and animals and contain multiple B- and T-cell epitopes that are able to elicit both neutralizing antibody response and T-cell mediated immunity [25]. Therefore, OMPs are likely the most successful candidate antigens for potential subunit vaccines. An ideal peptide-based vaccine should also contain both B- and T-cell epitopes. In our previous research, we identified six B- and T-cell combined immunodominant epitopes in the OMPs OmpL1, LipL32 and LipL21 from *Leptospira*, and tested whether they were able to induce specific immune responses [21, 22]. Considering the diversity among *Leptospira* serovars, these epitopes fragments are much conserved and show a high degree of homology among 15 serovar/serogroup standard reference strains of *Leptospira* in China. These six selected epitopes are only about 20 amino acids in length, which complicates mass production and makes them susceptible to host hydrolysis mechanisms. Therefore, in the current study, a gene fragment was synthesized that encoded all six epitopes separated by a four glycine spacer and which was codon optimized for expression in *E. coli*. This gene fragment was cloned as four consecutive copies in an expression vector and produced a 64 kD fusion protein. This recombinant protein was expressed in the soluble fraction, which is important to maintain its antigenicity and immunogenicity.

Antibodies are a major contributor to the phagocytosis and destruction of pathogenic *Leptospira* by macrophages [26]. Our r4R protein showed a strong immunogenicity in guinea pigs, which resulted in the production of high levels of specific antibodies. This is essential for the killing of *Leptospira* through complement-mediated lysis or through opsonin-dependent phagocytosis. To confirm that antibodies were reactive with a variety of leptospiral serovars, the MAT test was used, which is considered as the “golden standard” in the serodiagnosis of leptospirosis and the serological classification of *Leptospira* species. Our results revealed that the antisera obtained after immunization with r4R were able to agglutinate all standard reference strains of *Leptospira* in China, with MAT titers up to 1:320. Therefore, we expect that antibodies raised against our r4R protein are able to provide cross-protection against a wide variety of *Leptospira* serovars, although this still requires further confirmation using animal experiments.

Protective immunity to *Leptospira* is highly serovar-specific, which points to a dominant role for humoral immunity in the protection against *Leptospira* infection. However, recently it was shown that during an infection with *Leptospira*, cellular immunity still plays an important role in the control of disease [27]. Immunization with our r4R multi-epitope chimeric protein resulted in a dominant Th1 response, which was reflected by the yield of antigen-specific IgG subtypes (IgG2a > IgG1). A Th1 response generally induces IgG2a expression, while a Th2 response results predominantly in IgG1 production [28]. Besides mediating intracellular killing of a variety of pathogenic microorganisms, Th1 cells also stimulate B cells to produce antibodies that are important for mucosal immunity, such as IgM, IgG2a and IgA [29, 30], which is regulated via secretion of IFN-γ and IL-2 and via expression of CD40L [31]. Cytokine measurements in the culture supernatant of splenocytes from immunized guinea pigs, after stimulation with our r4R protein, showed high induction of IFN-γ compared with IL-4, which supports that the r4R multi-epitope chimeric protein vaccine is biased towards a Th1-type response.

Severe leptospirosis is typically associated with an overactive host response that damages its own tissues, such as the liver, kidney and lung, which can furthermore lead to hemorrhages. Guinea pigs are susceptible to *Leptospira* infection and frequently present these symptoms. In order to determine the ability of our r4R protein to induce a protective immune response against a *Leptospira* infection, guinea pigs were used as an infection model. Compared with unvaccinated guinea pigs, immunized animals showed increased survival, reduced tissue damage, decreased colonization of the kidneys and reduced shedding of *Leptospira* in the urine. However, immunized guinea pigs also showed some inflammatory reactions, such as edema symptoms. This may be the result of increased IFN-γ production after immunization, which can cause inflammation or a delayed type hypersensitivity [32].

## Conclusions

This study showed that the chimeric multi-epitope protein r4R is a promising vaccine candidate for the development of a universal vaccine against *Leptospira* infections. Immunized guinea pigs showed a strong antigen-specific humoral and cellular immune response, a reduction in tissue damage and colonization and protection against a lethal *Leptospira* infection. Thus, the multi-epitope chimeric r4R vaccine, containing both T- and B-cell epitopes, may represent an effective antigen to increase the efficacy of vaccines against leptospirosis.

## Methods

### Bacterial strains, media and plasmids

*Escherichia coli* host strains DH10B and BL21 (DE3) and the plasmids pBacPAK8 and pET-28a (+) were maintained in the lab. The pGEM-T easy vector was obtained from Promega (Wisconsin, USA). Endonucleases and the DNA ladder were bought from TaKaRa Bio Co., Ltd (Dalian, China). The protein molecular weight marker, lymphocyte separation medium (guinea pig), mitomycin and the CCK-8 kit were purchased from Beyotime Institute of Biotechnology (Jiangsu, China). All chemicals were of analytical grade. The secondary antibody-enzyme conjugates used in this study were obtained from Jackson ImmunoResearch (Pennsylvania, USA) or Santa Cruz (California, USA). The UltraEAL Western Blot Detection System was purchased from Shanghai Generay Biotech Co., Ltd (Shanghai, China). Sera from rabbits immunized with inactivated *L. interrogans* serovar Icterohaemorrhagiae Lai were maintained in our lab [16, 24, 33]. *Leptospira* strains are kept in our lab and cultured in EMHJ medium at 28 °C. New Zealand white rabbits were purchased from the Laboratory Animal Center of Zhejiang University. Three-week-old guinea pigs were purchased from Vital River Laboratories (Beijing, China) and screened by the microscopic agglutination test to ensure that they were free of preexisting antibodies to *Leptospira*.

### Design, expression and purification of chimeric peptide

Based on our previous studies, six OMP epitopes were selected, which each were 11–27 amino acids in length (Additional file 2) [21, 22]. Recombinant plasmids were constructed and target proteins were expressed and purified following methods published previously [34]. Briefly, a gene encoding six selected immunodominant epitopes, which were spaced by a tetraglycine linker and codon-optimized for *E. coli* translation, was synthesized. In the 5′ end, a *Bam*HI site was designed, and in the 3′ end, a *Bgl*II and an *Eco*RI site were designed and spaced by the terminator sequence TAA. Through four time consecutive cloning of the *Bam*HI/*Eco*RI-digested gene into its *Bgl*II/*Eco*RI site, the synthesized gene was repeated in four copies in the plasmid pBacPAK8-4R as a single gene. The multi-epitope gene was subsequently cloned into the *Bam*HI/*Eco*RI site of pET28a (pET28a-4R). Plasmid DNA of pET28a-4R was transformed into *E. coli* BL21 (DE3) for protein expression. The recombinant protein was expressed and purified using Ni-NTA Superflow resin (Biocolors, Shanghai, China). Possible endotoxin contamination from *E. coli* BL21 (DE3) was removed from the purified protein using the Detoxi-Gel Endotoxin Removal kit (Thermo Scientific, USA) [35]. Using LPS from *E. coli* serotype O111:B4 (Sigma) as control, a limulus test was carried out to detect LPS in the purified protein samples according to the manufacturer’s protocol.

### SDS-PAGE and Western blot for the detection of chimeric antigens

To detect the recombinant 4R protein with antibodies present in the serum of rabbits immunized with inactivated *L. interrogans* cells, the purified target protein was run on a 10 % SDS-PAGE gel, along with appropriate controls and prestained protein markers. The proteins were subsequently transferred to a polyvinylidene fluoride membrane (PVDF, Millpore) following Bio-Rad Protocols. The membrane was incubated in 1 × TBST (Tris buffered saline; 0.1 % Tween 20, pH 7.2) with 6 % newborn bovine serum for 1 h at 37 °C, washed three times with 1 × TBST and further incubated with rabbit anti-*Leptospira* serum (1:1 000 dilution) for 1 h at 37 °C. The membrane was washed three times with 1 × TBST, and incubated with a goat anti-rabbit IgG-HRP conjugate secondary antibody (1:5 000 dilution) for a further 1 h at 37 °C. Chemiluminescent detection was performed using Lumigen-PS according to the manufacturer’s protocol (GE) and blots were exposed against X-ray films for relevant time periods.

### Immunization of guinea pigs with purified recombinant 4R (r4R) protein and detection of the antibody response

For the safety test, guinea pigs (150 g ± 3 g, aged 3 weeks), were immunized with 200 μg r4R protein that had been pre-mixed with aluminum hydroxide (1.0 mg/ml, Sigma, USA). Administration was performed subcutaneously in the four limbs and an equivalent booster dose was given 14 days after the initial immunization. After 7–10 days, guinea pigs were sacrificed for further experiments. PBS immunized guinea pigs following the same procedure were used as control.

The immunogenicity of the r4R protein was analyzed by Western blot and ELISA. Blood collected from immunized guinea pigs was allowed to clot overnight, centrifuged at 4500 rpm for 10 min and the sera were collected and stored at −20 °C for further analysis. Western blot analysis was carried out as described above using a 1:150 dilution of sera from r4R-immunized animals and a 1:5000 dilution of the goat anti-guinea pig IgG-HRP conjugate secondary antibody.

For ELISA analysis, 96-well ELISA plates were coated with 0.5 μg/well of *L. interrogans* strain Lai diluted in 0.1 M Na_2_HPO_4_ (pH 9.0). After incubation overnight at 4 °C, the plates were washed three times with PBST (phosphate-buffered saline containing 0.05 % Tween 20, pH 7.4), blocked at 37 °C for 1 h with 100 μl of 1 % bovine serum albumin (BSA) in PBS, and washed three times with PBST. One hundred microliter optimal diluted (1:100) serum sample was added to each well and incubated at 37 °C for 2 h. After washing, 100 μl of goat anti-guinea pig IgG1 or IgG2a conjugated to horseradish peroxidase (ICLLab) diluted (1:100) with 1 % BSA in PBST was added to plates and incubated at 37 °C for 1 h. Following washing, 100 μl of concentrated 3,3′,5,5′-tetramethylbenzidine substrate was allowed to react with the HRP for 15 min at 37 °C in the dark. The color reaction was stopped by adding 100 μl of 2 M H_2_SO_4_, and the absorbance at a wavelength of 450 nm was read in a microplate reader.

MAT was used to detect the agglutination of immune sera with standard reference strains of *Leptospira* in China [5]. Briefly, the sera from immunized guinea pigs were diluted in a gradient (1:20, 1:40, 1:80, 1:160, 1:320 and 1:640) in normal saline and subsequently mixed with the same volume of *Leptospira* cultures. After incubated at 37 °C for 1 h, agglutination was determined by dark-field microscopy. The agglutination titer was determined as the highest dilution of serum which causes 50 % of the *Leptospira* to agglutinate compared with the negative control.

### LD_50_ determination and challenge experiments

*L. interrogans* strain Lai was propagated for 3 weeks in EMJH medium until dense cultures were observed using dark-field microscopy. The *Leptospira* were washed three times by centrifugation at 12,000 rpm for 15 min and resuspended in PBS. The suspension was then diluted to concentrations ranging from 1 × 10^7^ to 1 × 10^10^ cells/ml. To calculate the 50 % lethal dose (LD_50_), guinea pigs were divided into five groups, each containing six guinea pigs. Four of the groups were given different concentrations of fresh *Leptospira* (10^7^, 10^8^, 10^9^ and 10^10^ cells/ml)) intraperitoneally in 1 ml quantities. The animals of the control group were administered with EMJH. The animals were monitored twice daily for up to 21 days post challenge to record for signs of terminal disease (moribund), which was counted as dead. The surviving animals were recorded for calculating the LD_50_ using the probit analysis [36]. In the vaccine trial, the guinea pigs were divided into three groups, each with five animals. Animals from A group were used as unvaccinated healthy controls. Animals from group B were immunized with 200 μg r4R protein supplemented with aluminum hydroxide (1.0 mg/ml. Sigma, USA) as adjuvant. Booster doses of the same vaccine were given at 2 week intervals. Animals from group C were immunized with PBS and used as control animals. The guinea pigs in group B and C were challenged intraperitoneally with a 2 × LD_50_ of low-passage *L. interrogans* strain Lai 42 days after the initial immunization. The animals were monitored twice daily to observe for clinical symptoms. Urine samples, which were collected at weekly intervals following challenge, were processed for the detection of *Leptospira*. All animals were sacrificed and blood was collected from the eyeground for antibody detection. The tissues, including liver, kidney, spleen and lungs were collected aseptically for further analysis. The protective efficacy of the chimeric protein was replicated three times.

The kidneys of infected animals were homogenized in EMJH medium and subsequently transferred into 5 ml EMJH medium. After incubation at 30 °C for 28 days, the culture samples were monitored for growth. A tungsten lamp was used to determine the turbidity of the EMJH medium and after Fontana silver staining, the samples were observed using an optical microscope.

### Histopathology

Tissues from guinea pigs were immersed in 10 % neutral formaldehyde solution for 48 h, and then rinsed, dehydrated and embedded in paraffin. Tissue sections were stained with hematoxylin and eosin (H&E) using standard histological techniques, and examined by light microscopy [8, 37].

### CD4^+^ T lymphocyte proliferative response and cytokine evaluation

Spleens were aseptically removed from immunized guinea pigs and mechanically homogenized with a syringe plunger. The splenic T lymphocytes were isolated using lymphocyte separation medium (guinea pig) according to the manufacturer’s protocols, and the cells were suspended in complete RPMI-1640 media (RPMI-1640, 10 % fetal bovine serum, 2 mM glutamine, 50 U of penicillin/ml, 50 μg of streptomycin/ml, 50 μM 2-mercaptoethanol, and 25 mM HEPES).

Lymphocyte proliferation was evaluated using the CCK-8 assay kit as described previously [22]. Aliquots of 5 × 10^5^ splenic lymphocytes were dispensed into 96-well flat-bottomed microtiter plates and incubated at 37 °C with 5 % CO_2_ for 7 days in the presence of 5 μg pokeweed mitogen/ml and 2 μg purified r4R protein. Cells suspended in PBS were used as negative control, and the mitogen Concanavalin A (ConA, at a final concentration of 1 μg/ml) was used as positive control.

To determine the cytokine secretion pattern and evaluate Th cell polarization, cytokine levels in the supernatant of the cell culture (100 μl each) were evaluated using guinea pig IFN-γ or IL-4 ELISA kits (Westang, Shanghai). The cytokine levels were calculated by titration of supplied calibrated cytokine standards. All assays were performed as suggested by the manufacturer. The level of cytokines in the medium was calculated for each sample by subtracting the corresponding cytokine concentration produced in the control wells without antigen.

### Statistical analysis

Figures represent data from three independent experiments, and the data were expressed as mean ± standard deviation (S.D.). Microsoft office Excel was used to analyze variance and identify significant differences.

## Additional files

Additional file 1: Figure. Sequence of the synthetized gene fragment and encoded protein. The endonuclease sites are underlined. The tetraglycine linkers are boxed. (PPT 208 kb)
Additional file 2: Table. Sequences of immunodominant epitope used in this report. (DOC 30 kb)
