# Supplementary figures and images for: Chimeric epitope vaccine against Leptospira interrogans infection and induced specific immunity in guinea pigs
Source: BMC Microbiol. 2016 Oct 14;16:241. doi: 10.1186/s12866-016-0852-y (PMC5064800; doi:10.1186/s12866-016-0852-y)

## Slide 1
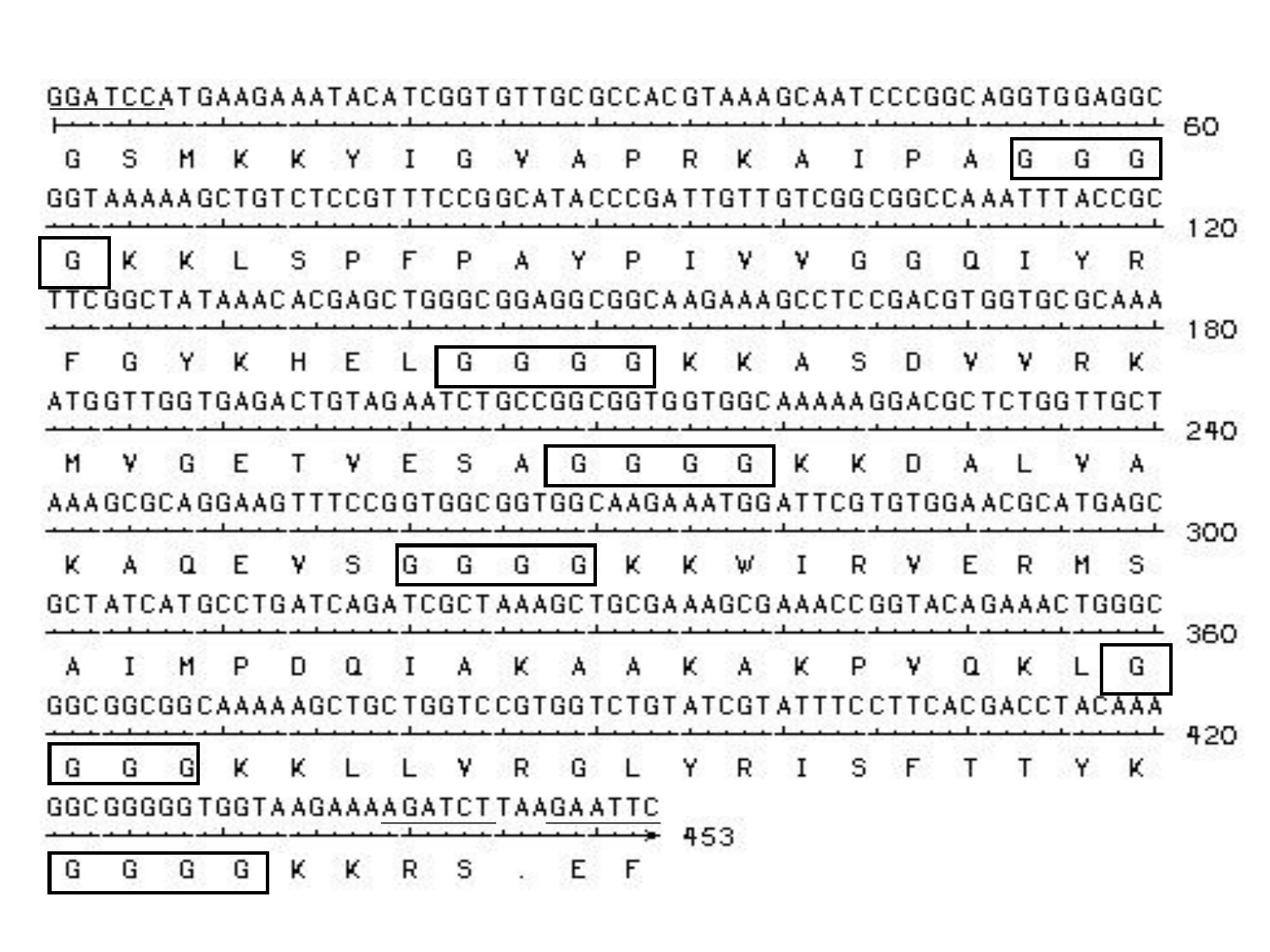

Supplement: Additional file 1: Figure. — Sequence of the synthetized gene fragment and encoded protein. The endonuclease sites are underlined. The tetraglycine linkers are boxed. (PPT 208 kb) [file 12866_2016_852_MOESM1_ESM.ppt]
