# Supplementary material for: Chimeric epitope vaccine against Leptospira interrogans infection and induced specific immunity in guinea pigs
Source: BMC Microbiol. 2016 Oct 14;16:241. doi: 10.1186/s12866-016-0852-y (PMC5064800; doi:10.1186/s12866-016-0852-y)
Supplement: Additional file 2: Table. — Sequences of immunodominant epitope used in this report. (DOC 30 kb) [file 12866_2016_852_MOESM2_ESM.doc]

Table . Sequences of immunodominant epitope used in this report

| No. | Protein | Location | Sequence (N’-C’) |
| --- | --- | --- | --- |
| 1 | OmpL1 | 87-98 | YIGVAPRKAIPA |
| 2 |  | 173-191 | SSIVIPAAVGIKLNVTEDA |
| 3 | LipL32 | 133-160 | WIRVERMSAIMPDQIAKAAKAKPVQKL |
| 4 |  | 201-218 | KKLLVRGLYRISFTTYK |
| 5 | LipL21 | 97-112 | ASDVVKKMVGETVESA |
| 6 |  | 176-184 | DALVAKAQEVS |
